# Multilocus Phylogenetic Identification and Fruit Pathogenicity of *Lasiodiplodia* Isolates Obtained from Mango Branches with Dieback and Fruits with Stem-End Rot in Mexico

**DOI:** 10.3390/jof12050370

**Published:** 2026-05-16

**Authors:** Juan Manuel Tovar-Pedraza, Guillermo Estrada-Arroyo, Rafael Macedo-Arzate, Sami J. Michereff, Kamila C. Correia, Santos Gerardo Leyva-Mir, José Antonio Mora-Aguilera, Moisés Camacho-Tapia, Guillermo Márquez-Licona, Alma Rosa Solano-Báez

**Affiliations:** 1 Laboratorio de Fitopatología, Centro de Investigación en Alimentación y Desarrollo, Subsede Culiacán, Culiacán 80110, Mexico; juan.tovar@ciad.mx; 2 Departamento de Parasitología Agricola, Universidad Autónoma Chapingo, Texcoco 56230, Mexico; memoestradaa@hotmail.com (G.E.-A.); rafael199004.rma@gmail.com (R.M.-A.); lsantos@correo.chapingo.mx (S.G.L.-M.); moises.camachotapia@gmail.com (M.C.-T.); 3 Centro de Ciências Agrárias e da Biodiversidade, Universidade Federal do Cariri, Crato 63130-025, Brazil; sami.michereff@ufca.edu.br (S.J.M.); kamila.correia@ufca.edu.br (K.C.C.); 4 Fitopatología, Colegio de Postgraduados, Campus Montecillo, Texcoco 56230, Mexico; aguilera@colpos.mx; 5 Centro de Desarrollo de Productos Bióticos, Instituto Politécnico Nacional, Yautepec 62731, Mexico; gmarquezl@ipn.mx

**Keywords:** *Mangifera indica*, *Botryosphaeriaceae*, multilocus phylogeny, stem-end rot, dieback, aggressiveness assay

## Abstract

Mango (*Mangifera indica* L.) represents one of the most significant fruit crops cultivated across multiple regions of Mexico. In recent years, cases of stem-end rot and dieback have been observed in mango-producing areas. This research aimed to characterize the diversity of *Lasiodiplodia* species associated with these symptoms, determine their geographic distribution in five Mexican states, and evaluate their pathogenicity on mango fruits. During 2014, samples exhibiting dieback and stem-end rot symptoms were collected from 27 commercial orchards located in five states, resulting in the obtention of 87 *Lasiodiplodia* isolates. From these, 36 representative isolates were selected and identified through phylogenetic analyses (ITS, *tef1-α*, *tub2*), employing the Maximum Likelihood and Bayesian Inference approach. Eight *Lasiodiplodia* species were resolved: *L. brasiliense*, *L. laeliocattleyae*, *L. subglobosa*, *L. theobromae*, *L. iraniensis*, *L. mexicanensis*, *L. hyalina*, and *L. pseudotheobromae*. Among them, *L. brasiliense*, *L. laeliocattleyae*, *L. subglobosa*, *L. iraniensis*, *L. mexicanensis*, and *L. hyalina* are reported for the first time in association with mango tissues in Mexico. Pathogenicity tests conducted on detached mango fruits using the mycelial plug inoculation method demonstrated that all species were capable of inducing necrotic lesions. However, *L. laeliocattleyae* and *L. brasiliense* exhibited the highest levels of aggressiveness, while *L. mexicanensis*, *L. hyalina*, and *L. pseudotheobromae* were the least aggressive.

## 1. Introduction

The mango (*Mangifera indica* L.) ranks among the top five fruit crops of greatest economic relevance globally, cultivated extensively throughout tropical and subtropical regions [1,2]. Mexico stands as the world’s fifth-largest producer and the leading exporter of this fruit [3]. Although mango cultivation occurs in 23 Mexican states, production is mainly concentrated in ten: Sinaloa, Guerrero, Nayarit, Chiapas, Oaxaca, Michoacán, Jalisco, Veracruz, Colima, and Tamaulipas [3].

The Botryosphaeriaceae family comprises 24 genera [4,5] and 280 species [6,7] of a diverse group of endophytic, necrotrophic pathogens, and saprophytic fungi that colonize the inner tissues of woody plants and are increasingly associated with dieback events globally [8,9,10]. Several Botryosphaeriaceae species have been previously associated with mango diseases, including *Botryosphaeria* spp. [11,12,13], *Fusicoccum* spp. [13,14], *Neofusicoccum* spp. [11,12,14,15,16], *Neoscytalidium* spp. [13], and *Lasiodiplodia* spp. [11,14,15,17,18,19,20,21,22].

Accurate identification of plant pathogenic species is essential for elucidating their epidemiological behavior and for designing efficient management and control strategies [23]. The distinction of *Lasiodiplodia* species by morphological characterization is not reliable, and it is necessary to use DNA sequence data, preferably combining sequences from multiple loci such as ITS, *tef1-α*, and *tub2*, for accurate identification [17,24,25,26,27,28,29].

At present, no fewer than ten *Lasiodiplodia* species have been reported in association with mango tissues worldwide [30]. Nevertheless, in Mexico, only *L. theobromae* and *L. pseudotheobromae* have been documented as causal agents of stem-end rot and dieback in mango, respectively [16]. This study aimed to elucidate the diversity of *Lasiodiplodia* species linked to these diseases through phylogenetic analysis, assess their geographic distribution across mango-producing regions, and evaluate their aggressiveness on mango fruits.

## 2. Materials and Methods

### 2.1. Field Surveys

From May to November 2014, surveys were conducted in 27 commercial mango orchards distributed in the states of Sinaloa, Guerrero, Veracruz, Chiapas, and Tabasco, in Mexico. Mango branches with dieback, as well as fruits with stem-end rot symptoms, were collected from various mango cultivars (Manila, Tommy Atkins, Haden, Ataulfo, Kent, and Keitt).

### 2.2. Isolation, Purification, and Preservation of Fungi

Tissue fragments taken from the transition zone between necrotic and healthy areas of branches and fruits were surface-disinfested in 1% sodium hypochlorite for 1 min, rinsed three times with sterile distilled water, and dried on sterile absorbent paper. The disinfected pieces were then placed on Petri dishes containing potato dextrose agar (PDA; Difco^®^, Sparks, MD, USA) supplemented with 0.5 g L^−1^ streptomycin sulfate (Sigma-Aldrich^®^, St. Louis, MO, USA) and incubated in darkness at 25 °C. Pure cultures were obtained by transferring hyphal tips from the actively growing margins of colonies onto fresh PDA. All isolates used in this investigation were preserved in the Culture Collection of Phytopathogenic Fungi “Professor Maria Menezes” (CMM), Universidade Federal Rural de Pernambuco, Recife, Brazil, under accession numbers CMM3106–CMM3201.

### 2.3. DNA Extraction, PCR Amplification, and Sequencing

For each *Lasiodiplodia* isolate, a small portion of aerial mycelium was collected from six-day-old cultures using a sterile 10 µL pipette tip. Genomic DNA from 87 isolates was extracted with the Multisource Genomic DNA Miniprep Kit (Axygen Scientific^®^, Union City, CA, USA) according to the manufacturer’s protocol. Amplification of partial fragments of the *tef1-α* EF1-688F/EF1-1251R [31]. To confirm species, amplification of the internal transcribed spacer (ITS) region and partial fragments of the *tub2* genes was performed using the primer pairs ITS1/ITS4 [32] and Bt2A/Bt2B [33], respectively. Each 50 µL PCR reaction contained 21 µL of PCR-grade water, 4 µL of DNA template, 2.5 µL each primer (10 μM of each primer), and 20 µL of 2× PCR Master Mix [0.05 U µL^−1^ Taq DNA polymerase, reaction buffer, 4 mM MgCl_2_, and 0.4 mM of each dNTP] (Thermo Scientific, Waltham, MA, USA). Amplifications were carried out in a Biocycler MJ96 thermocycler (Applied Biosystems, Carlsbad, CA, USA). PCR products were resolved on 1.5% agarose gels stained with ethidium bromide (0.5 µg mL^−1^) and visualized under ultraviolet light. The resulting amplicons were purified using the AxyPrep PCR Purification Kit (Axygen, Union City, CA, USA) and bidirectionally sequenced by Macrogen^®^ (Seoul, Republic of Korea) employing the same primers used in the amplification reactions.

### 2.4. Phylogenetic Analysis

Chromatograms obtained from sequencing the ITS region and partial *tef1-α* and *tub2* gene fragments were examined and assembled using the Staden Package version 2.0 [34]. Sequence alignments were generated with ClustalX v1.83 [35] integrated in the MEGA v7.0 software [36], with manual adjustments applied when necessary to improve alignment quality. Reference sequences of *Lasiodiplodia* species retrieved from GenBank were incorporated into the alignments for comparative analysis. Phylogenetic relationships were inferred using the Maximum Likelihood (ML), first for the *tef1-α* locus, selecting isolates that represented the greatest diversity of possible species. The concatenated datasets (ITS, *tef1-α*, and *tub2*) of the selected isolates were then analyzed. The resulting sequences were compared with type and reference isolates based on the most recent taxonomic studies of *Lasiodiplodia*. ML analyses were carried out in IQ-TREE v2.3.5 [37], with the optimal nucleotide substitution models determined by ModelFinder V2.3.5 [38,39]. Each ML run included 1000 bootstrap replications using the ultrafast bootstrapping algorithm implemented in IQ-TREE. Additionally, a Bayesian Inference (BI) analysis was conducted in MrBayes v.3.1.2 [40], using nucleotide substitution models selected by the BIC criterion (HKY + G for ITS and *tub2* and GTR + I + G for *tef1-α*). Markov Chain Monte Carlo (MCMC) simulations with 2 × 10^6^ generations were performed to estimate posterior probabilities. Trees were sampled every 1000 generations, and 25% of the trees produced during the burn-in phase were discarded.

### 2.5. Pathogenicity and Aggressiveness on Detached Fruits

Pathogenicity assays were performed on detached fruits of the mango cv. Ataulfo at the second color-break stage of ripening. The fruits were first washed thoroughly under running tap water, then surface-sterilized in 1% sodium hypochlorite for 3 min, immersed in 70% ethanol for 1 min, rinsed twice with sterile distilled water, and air-dried inside a laminar flow hood. Each fruit was wounded to a depth of approximately 3 mm using a sterile toothpick, and a 5 mm mycelial disk taken from the actively growing edge of a 5-day-old PDA culture was placed onto each wound. For control treatments, agar plugs without fungal growth were applied to ten fruits. All fruits were incubated at 25 °C under a 12 h light/12 h dark cycle in plastic trays lined with two layers of moistened sterile paper towels and sealed within plastic bags to maintain humidity. Three days after inoculation, disease severity was quantified by measuring lesion diameters to assess isolate aggressiveness. Each isolate was tested on three fruits, and the experiment was conducted twice. Differences in aggressiveness among *Lasiodiplodia* species were analyzed using one-way analysis of variance (ANOVA), and mean separations were determined by Fisher’s LSD test at a 5% significance level with SAS software version 9.1 (SAS Institute, Cary, NC, USA).

## 3. Results

### 3.1. Fungal Isolates

A total of 87 *Lasiodiplodia* isolates were obtained from mango symptomatic tissues (branches and fruits) collected from 27 orchards distributed in five states of Mexico (Sinaloa, Guerrero, Veracruz, Chiapas, and Tabasco).

### 3.2. Phylogenetic Analysis

**Figure 1:**
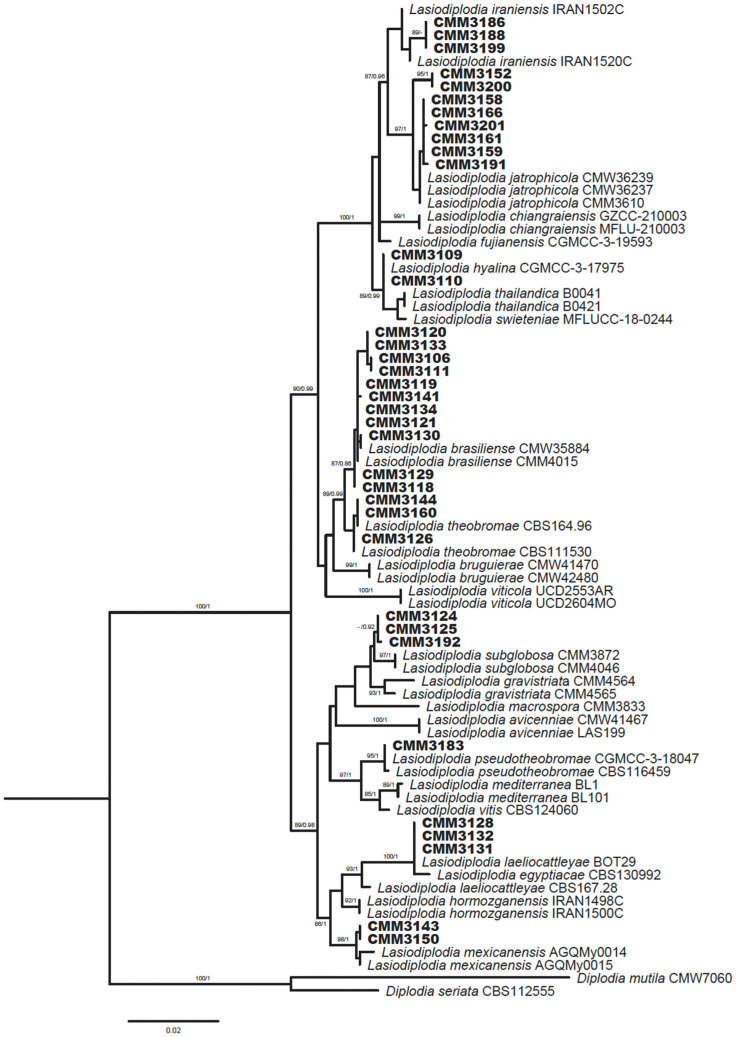
Phylogenetic tree of *Lasiodiplodia* species based on concatenated sequences of ITS region, *tef1-α*, and *tub2* genes using the Maximum Likelihood method. The isolates from this study are indicated in bold. Maximum Likelihood bootstrap values above 85 with 1000 replications, followed by Bayesian posterior probabilities, are shown at the branch. The tree is rooted to *Diplodia seriata* (CBS112555) and *D. mutila* (CMW7060).

All 87 isolates were previously identified as *Lasiodiplodia* spp. based on a phylogenetic analysis of the *tef1-α* gene. To confirm the species identity of the isolates, the ITS region and partial *tub2* gene sequences were obtained for 36 isolates representing isolates (Table 1). Multilocus sequence analysis was conducted using concatenated sequences of the three genetic markers, and sequences of ex-type isolates of *Lasiodiplodia* species from GenBank were included in the analysis together with isolates obtained in this study (Table 2). The ITS and *tef1-α* genetic alignments contained 77 taxa, and the *tub2* genetic alignment contained 71 taxa, including *Diplodia mutila* (CMW7060) and *Diplodia seriata* (CBS112555) as outgroups. According to multilocus analysis, the 36 representative isolates were distributed among eight previously described *Lasiodiplodia* species (Figure 1). These species presented significant support in the ML analysis (85–100% SH-alrt bootstrap support) and in the BI analysis (0.86–1 posterior probability). Eleven isolates were grouped within each of the *L. iraniensis* and *L. brasiliense* clades, while three isolates clustered in each of the *L. theobromae*, *L. subglobosa*, and *L. laeliocattleyae* clades. Two isolates were grouped within each of the *L. hyalina* and *L. mexicanensis* clades. While the isolate CMM3183 clustered with *L. pseudotheobromae*.

### 3.3. Distribution of Lasiodiplodia Species

**Figure 2:**
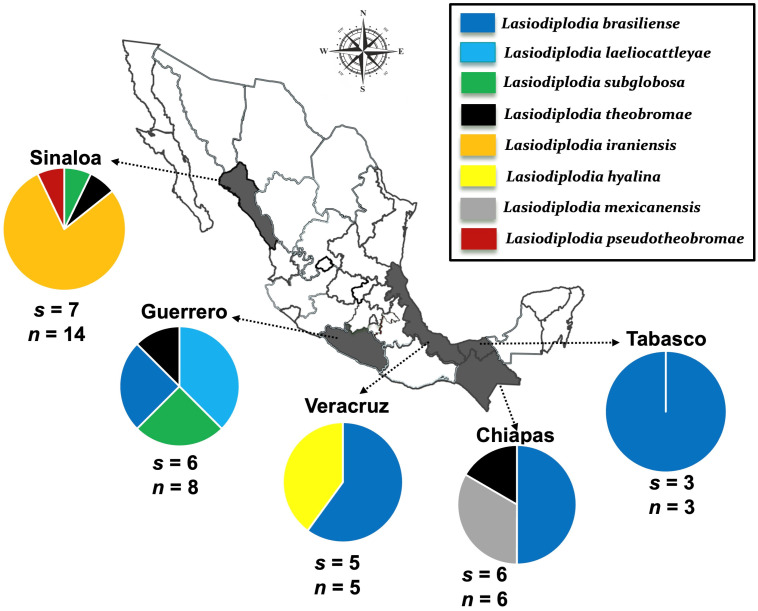
Collection sites of eight *Lasiodiplodia* spp. associated with mango dieback in five states in Mexico. Circles represent association frequency of each species with mango trees exhibiting symptoms of dieback in each population sampled; “*s*” is the number of commercial orchards sampled in each population and “*n*” is the number of isolates analyzed in each population.

Out of the 36 isolates phylogenetically identified in this study, *L. brasiliense* and *L. iraniensis* were the most frequently isolated species, each representing 30.5% of the isolates from symptomatic mango tissues. *Lasiodiplodia theobromae* (8.3%), *L. subglobosa* (8.3%), *L. laeliocattleyae* (8.3%), *L. mexicanensis* (5.5%), *L. hyalina* (5.5%), and *L. pseudotheobromae* (2.7%) were identified less frequently. The distribution of *Lasiodiplodia* species varied among the mango-growing states in Mexico (Figure 2). *Lasiodiplodia brasiliense* was the most widely distributed species, being found in four out of the five states. The states of Sinaloa and Guerrero exhibited the highest species diversity, with four species each, followed by Chiapas with three species and Veracruz with two. In contrast, Tabasco exhibited the lowest species diversity, with only *L. brasiliense* detected.

### 3.4. Pathogenicity and Aggressiveness on Fruits

**Figure 3:**
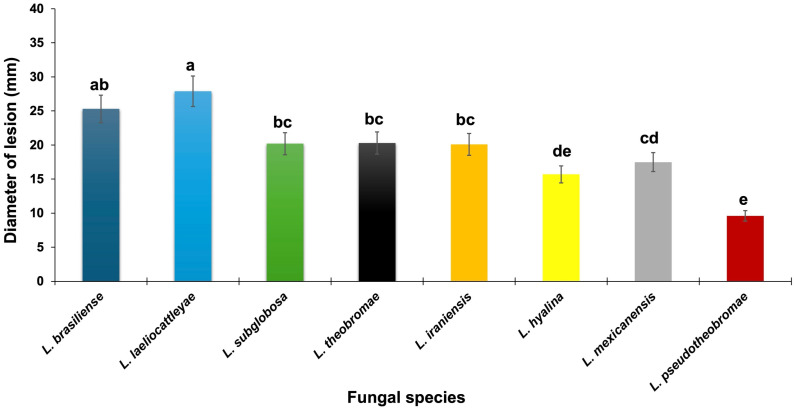
Necrotic lesion length (mm) of eight *Lasiodiplodia* species inoculated in detached mango fruit. Data is lesion diameter measured three days after inoculation with mycelium-colonized agar plugs inserted into wounded mango fruits. Bars above columns are the standard errors of the means. Columns with the same letter do not differ significantly, according to Fisher’s LSD test (*p* ≤ 0.05).

Three days after inoculation, all isolates representing the eight *Lasiodiplodia* species identified in this study proved pathogenic on mango fruits when tested using the mycelial plug method. Inoculated fruits developed irregular, necrotic lesions on the pericarp, whereas no symptoms were observed on the control fruits. Fungal reisolation from symptomatic tissues consistently yielded *Lasiodiplodia* spp., while none were recovered from control fruits, thereby satisfying Koch’s postulates. Since no significant differences were detected between the two experimental runs (*p* = 0.05), the data were pooled for subsequent statistical analyses. The combined results of both trials are presented in Figure 3, showing the mean lesion diameters produced by each *Lasiodiplodia* species.

Significant variation (*p* ≤ 0.05) in lesion size was observed among the species tested. The most extensive lesions (>28 mm in diameter) were caused by *L. laeliocattleyae*, indicating its high aggressiveness, followed by *L. brasiliense* (25 mm). *Lasiodiplodia iraniensis*, *L. mexicanensis*, *L. subglobosa*, *L. theobromae*, and *L. hyalina* displayed moderate aggressiveness, producing lesions ranging from 15 to 21 mm, whereas *L. pseudotheobromae* was the least aggressive, with mean lesions smaller than 10 mm (Figure 3).

## 4. Discussion

This study showed findings of eight *Lasiodiplodia* species associated with mango trees in Mexico. The phylogenetic analyses of combined ITS, *tef1-α*, and *tub2* sequence datasets revealed that *L. brasiliense*, *L. laeliocattleyae*, *L. subglobosa*, *L. theobromae*, *L. iraniensis*, *L. mexicanensis*, *L. hyalina*, and *L. pseudotheobromae* were associated with symptoms of dieback and stem-end rot in mango orchards. Thus, this study represents the first report of *L. brasiliense*, *L. laeliocattleyae*, *L. subglobosa*, *L. iraniensis*, *L. mexicanensis*, and *L. hyalina* associated with mango tissues in Mexico. Additionally, this is the first global report of *L. subglobosa*, *L. mexicanensis*, and *L. hyalina* as causal agents of mango diseases.

A comprehensive morphological characterization of the *Lasiodiplodia* isolates was not undertaken, since morphological features alone are unreliable for species delimitation within the Botryosphaeriaceae. Such features are useful primarily for distinguishing genera and for complementing molecular phylogenetic analyses when describing novel taxa [9,17,24,41,42]. The most robust approach for discriminating species in this family relies on DNA sequence data, ideally incorporating multilocus datasets from different genetic markers to ensure accurate identification [19,24,29,41,43]. The diversity of *Lasiodiplodia* species found in our study was consistent with the diversity reported from mango samples in Peru [19]. This is because both in Peru and Mexico, the species *L. brasiliense*, *L. laeliocattleyae*, *L. iraniensis*, *L. pseudotheobromae*, and *L. theobromae* were identified. However, the predominant species in Mexico were *L. brasiliense* and *L. iraniensis*, whereas in Peru it was *L. theobromae*.

Regarding the aggressiveness of isolates from different *Lasiodiplodia* spp., there was considerable variation between the species reported in Peru [19] and those in our study. This variation may be attributed to the different number of isolates evaluated per species, as well as to the fact that in the study conducted in Peru [19]; the aggressiveness was assessed on mango branches, whereas in our study, it was evaluated on mango fruits.

*Lasiodiplodia brasiliense* and *L. iraniensis* were the most frequently isolated pathogens causing diseases in mango orchards in our study. However, *L. brasiliense* was the most widely distributed species and was found in four of the populations analyzed. This fungal species has been previously reported to cause diseases in mango in Peru [19], China [44], Burkina Faso [45], and Ivory Coast [46].

*Lasiodiplodia iraniensis* has been frequently recovered from mango tissues exhibiting symptoms of dieback and stem-end rot in various regions around the world [11,15,19,20,22,47]. In our pathogenicity experiments, isolates of *L. iraniensis* showed high aggressiveness, which agrees with the results reported for *L. iraniensis* isolates obtained from mango in Brazil [20].

*Lasiodiplodia theobromae* is a common pathogen in mango, recorded in several countries [30]. Our findings on aggressiveness agree with those found by Marques et al. (2013) [13], Munirah et al. (2017) [20], and Rodríguez-Gálvez et al. (2017) [19], who determined that *L. theobromae* was more virulent than *L. pseudotheobromae* on mango.

*Lasiodiplodia laeliocattleyae* (syn. *Lasiodiplodia egyptiacae*) was previously reported as a causal agent of mango diseases in Peru [19]. It is important to note that this fungal species was the most aggressive among the eight species evaluated in our pathogenicity tests conducted in mango fruits.

*Lasiodiplodia pseudotheobromae* was the less common species among the isolates obtained in this study from symptomatic mango tissues in Mexico. This species has been reported to cause mango diseases in Australia [11], Brazil [22], Egypt [15], Malaysia [20], Peru [19], Pakistan [48], and Burkina Faso [45]. Results of our pathogenicity tests showed that *L. pseudotheobromae* was less aggressive than *L. laeliocattleyae.* However, Ismail et al. (2012) [15] and Marques et al. (2013) [22] determined that *L. pseudotheobromae* isolates were more aggressive than *L. laeliocattleyae* isolates when inoculated to mango seedlings and fruits, respectively.

In summary, this research demonstrates that mango production in Mexico, like in other fruit crops, is affected by a considerable diversity of *Lasiodiplodia* species. The outcomes of this study are of broad significance for understanding the etiology and epidemiology of the disease, as precise identification of closely related *Lasiodiplodia* taxa is essential for mapping their geographic distribution and preventing their dissemination to new cultivation areas. Moreover, the presence of this *Lasiodiplodia* species complex poses a serious challenge to the mango industry. Therefore, additional investigations focusing on their pathogenic impact, fungicide sensitivity, and comparative epidemiology are required to develop effective strategies for the integrated management of mango diseases including branch dieback and stem-end rot in Mexico.

## Figures and Tables

**Table 1:** *Lasiodiplodia* spp. isolates collected from branch dieback and stem-end rot of mango orchards in Mexico and included in this study.

Species	Isolate	Mango Cultivar	Organ	Locality	GenBank Accession No.		
					ITS	*Tef1α*	*Tub2*
*L. brasiliense*	CMM3106	Manila	Stem	Cazones, Veracruz	PV590619	PV575859	PV5954651
	CMM3111	Manila	Fruit	Cazones, Veracruz	PV590620	PV575860	PV5954652
	CMM3118	Manila	Stem	Cazones, Veracruz	PV590621	PV575861	PV5954653
	CMM3119	Haden	Fruit	Frontera, Tabasco	PV590622	PV575862	PV5954654
	CMM3120	Haden	Stem	Frontera, Tabasco	PV590623	PV575863	PV5954655
	CMM3121	Tommy Atkins	Stem	Frontera, Tabasco	PV590624	PV575864	PV5954656
	CMM3129	Tommy Atkins	Fruit	Cutzamala, Guerrero	PV590629	PV575865	PV5954649
	CMM3130	Tommy Atkins	Stem	Cutzamala, Guerrero	PV590630	PV575866	PV5954650
	CMM3133	Ataulfo	Stem	Tonala, Chiapas	PV590633	PV575867	PV5954646
	CMM3134	Ataulfo	Stem	Metapa, Chiapas	PV590634	PV575868	PV5954647
	CMM3141	Ataulfo	Stem	Tonala, Chiapas	PV590635	PV575869	PV5954648
*L. hyalina*	CMM3109	Manila	Fruit	Cazones, Veracruz	PV590617	PV582999	PV5954644
	CMM3110	Manila	Stem	Cazones, Veracruz	PV590618	PV583000	PV5954645
*L. iraniensis*	CMM3152	Kent	Stem	Ahome, Sinaloa	PV590639	PV583001	PV5954674
	CMM3158	Kent	Stem	Ahome, Sinaloa	PV590640	PV583006	PV5954675
	CMM3159	Kent	Stem	Ahome, Sinaloa	PV590641	PV583003	PV5954676
	CMM3161	Kent	Stem	Ahome, Sinaloa	PV590643	PV583007	PV5954677
	CMM3166	Kent	Stem	Ahome, Sinaloa	PV590644	PV583008	PV5954678
	CMM3186	Keitt	Stem	Ahome, Sinaloa	PV590646	PV583010	PV5954668
	CMM3188	Keitt	Stem	Ahome, Sinaloa	PV590647	PV583009	PV5954669
	CMM3191	Keitt	Stem	Ahome, Sinaloa	PV590648	PV583004	PV5954673
	CMM3199	Keitt	Stem	Ahome, Sinaloa	PV590650	PV583011	PV5954670
	CMM3200	Keitt	Stem	Ahome, Sinaloa	PV590651	PV583002	PV5954671
	CMM3201	Keitt	Stem	Ahome, Sinaloa	PV590652	PV583005	PV5954672
*L. laeliocattleyae*	CMM3128	Tommy Atkins	Stem	Cutzamala, Guerrero	PV590628	PV583015	PV5954663
	CMM3131	Tommy Atkins	Fruit	Cutzamala, Guerrero	PV590631	PV583016	PV5954664
	CMM3132	Tommy Atkins	Stem	Cutzamala, Guerrero	PV590632	PV583017	PV5954665
*L. mexicanensis*	CMM3143	Ataulfo	Fruit	Tonala, Chiapas	PV590636	PV583018	PV5954666
	CMM3150	Ataulfo	Stem	Metapa, Chiapas	PV590638	PV583019	PV5954667
*L. pseudotheobromae*	CMM3183	Keitt	Stem	Ahome, Sinaloa	PV590645	PV583020	PV5954679
*L. subglobosa*	CMM3124	Tommy Atkins	Stem	Cutzamala, Guerrero	PV590625	PV583012	PV5954658
	CMM3125	Tommy Atkins	Fruit	Cutzamala, Guerrero	PV590626	PV583013	PV5954655
	CMM3192	Keitt	Stem	Ahome, Sinaloa	PV590649	PV583014	PV5954657
*L. theobromae*	CMM3126	Tommy Atkins	Fruit	Cutzamala, Guerrero	PV590627	PV582996	PV5954661
	CMM3144	Manililla	Stem	Pijijiapan, Chiapas	PV590637	PV582997	PV5954662
	CMM3160	Kent	Stem	Ahome, Sinaloa	PV590642	PV58298	PV5954660

**Table 2:** GenBank accession numbers of DNA sequences of reference isolates used for phylogenetic analyses.

Species	Isolate Code	Host	Origin	GenBank Accession Number		
				ITS	*tef1-α*	*tub2*
*Diplodia mutila*	CMW7060	*Fraxinus excelsior*	Netherlands	CMW7060	AY236955	AY236904
*D. seriata*	CBS112555	*Vitis vinifera*	Portugal	AY259094	AY573220	------
*Lasiodiplodia avicenniae*	CMW41467	*Avicennia marina*	South Africa	KP860835	KP860680	KP860758
*L. avicenniae*	LAS199	*Avicennia marina*	South Africa	KU587957	KU587947	KU587868
*L. brasiliense*	CMM4015	*Mangifera indica*	Brazil	JX464063	JX464049	------
*L. brasiliense*	CMW35884	*A. madagascariensis*	Madagascar	KU887094	KU886972	KU887466
*L. bruguierae*	CMW41470	*Bruguiera gymnorrhiza*	South Africa	KP860832	KP860677	KP860755
*L. bruguierae*	CMW42480	*Bruguiera gymnorrhiza*	South Africa	KP860834	KP860679	KP860757
*L. chiangraiensis*	MFLUCC-21-0003	------	Thailand	MW760854	MW815630	MW815628
*L. chiangraiensis*	GZCC-21-0003	------	Thailand	MW760853	MW815629	MW815627
*L. fujianensis*	CGMCC-3-19593	*Vaccinium uliginosum*	China	MK802164	MK887178	MK816337
*L. gravistriata*	CMM4564	*Anacardium humile*	Brazil	KT250949	KT250950	------
*L. gravistriata*	CMM4565	*Anacardium* sp.	Brazil	KT250947	KT266812	------
*L. hormozganensis*	IRAN1500C	*Olea* sp.	Iran	GU945355	GU945343	KU887515
*L. hormozganensis*	IRAN1498C	*Mangifera indica*	Iran	GU945355	GU945344	KU887514
*L. hyalina*	CGMCC3.17975	*Acacia confusa*	China	KX499879	KX499917	KX499992
*L. iraniensis*	IRAN1502C	*Juglans* sp.	Iran	GU945347	GU945335	KU887517
*L. iraniensis*	IRAN1520C	*Salvadora persica*	Iran	GU945348	GU945336	KU887516
*L. iraniensis*	CMM3610	*Jatropha curcas*	Brazil	KF234544	KF226690	KF254927
*L. iraniensis*	CMW36237	*Adansonia digitata*	Mozambique	KU887121	KU886998	KU887499
*L. iraniensis*	CMW36239	*Adansonia digitata*	Mozambique	KU887123	KU887000	KU887501
*L. laeliocattleyae*	CBS 167.28	*Laeliocattleyae* sp.	Italy	KU507487	KU507454	------
*L. laeliocattleyae*	BOT 29	*Mangifera indica*	Egypt	JN814401	JN814428	------
*L. laeliocattleyae*	CBS130992	*Mangifera indica*	Egypt	JN814397	JN814424	KU887508
*L. macrospora*	CMM3833	*Jatropha curcas*	Brazil	KF234557	KF226718	KF254941
*L. mediterranea*	BL1	*Quercus ilex*	Italy	KJ638312	KJ638331	------
*L. mediterranea*	BL101	*Vitis* sp.	Italy	KJ638311	KJ638330	------
*L. mexicanensis*	AGQMy 0014	*Chamaedorea seifrizii*	Mexico	MW274151	MW604234	MW604243
*L. mexicanensis*	AGQMy0015	*Chamaedorea seifrizii*	Mexico	MW274150	MW604233	MW604242
*L. pseudotheobromae*	CBS116459	*Gmelina arborea*	Costa Rica	EF622077	EF622057	EU673111
*L. pseudotheobromae*	CGMCC-3-18047	*Pteridium* sp.	China	KX499876	KX499914	KX499989
*L. subglobosa*	CMM3872	*Jatropha curcas*	Brazil	KF234558	KF226721	KF254942
*L. subglobosa*	CMM4046	*Jatropha curcas*	Brazil	KF234560	KF226723	KF254944
*L. swieteniae*	MFLUCC-18-0244	*Swietenia mahagoni*	Thailand	MK347789	MK340870	MK412877
*L. thailandica*	B0041	*Phyllanthus acidus*	Thailand	KM006433	KM006464	------
*L. thailandica*	B0421	*Mangifera indica*	Thailand	KJ193637	KJ193681	------
*L. theobromae*	CBS111530	Unknown	Unknown	EF622074	EF622054	KU887531
*L. theobromae*	CBS164.96	Unknown	New Guinea	AY640255	AY640258	KU887532
*L. viticola*	UCD2553AR	*Vitis vinifera*	USA	HQ288227	HQ288269	HQ288306
*L. viticola*	USD2604MO	*Vitis vinifera*	USA	HQ288228	HQ288270	HQ288307
*L. vitis*	CBS 124060	*Vitis vinifera*	Italy	KX464148	KX464642	KX464917

MFLUCC: Mae Fah Luang University Culture Collection, Chiang Rai, Thailand; IRAN: Culture Collection of the Iranian Research Institute of Plant Protection, Tehran, Iran; CBS: Centraalbureau voor Schimmelcultures, Utrecht, Netherlands; GZCC: Guizhou Culture Collection, Guiyang, China; CMW: Forestry and Agricultural Biotechnology Institute, University of Pretoria, South Africa; CMM: Culture Collection of Phytopathogenic Fungi ‘Professora Maria Menezes’, Universidade Federal Rural de Pernambuco, Recife, Brazil; CGMCC: China General Microbiological Culture Collection, Beijing, China; BL: Personal number of B.T. Linaldeddu; USD: Phaff Yeast Culture Collection, Department of Food Science and Technology, University of California, Davis, USA.

## Data Availability

The original contributions presented in this study are included in the article. Further inquiries can be directed to the corresponding author.
